# Physiological and pathological high-frequency oscillations have distinct sleep-homeostatic properties

**DOI:** 10.1016/j.nicl.2017.02.018

**Published:** 2017-02-24

**Authors:** Nicolás von Ellenrieder, François Dubeau, Jean Gotman, Birgit Frauscher

**Affiliations:** a Montreal Neurological Institute and Hospital, McGill University, 3801 University Street, Montreal H3A 2B4, Québec, Canada; b Department of Medicine and Center for Neuroscience Studies, Queen's University, 18 Stuart Street, Kingston K7L3N6, Ontario, Canada

**Keywords:** Epilepsy, Intracerebral EEG, Polysomnography, High frequency oscillations, Sleep

## Abstract

**Objective:**

The stage of sleep is a known modulator of high-frequency oscillations (HFOs). For instance, high amplitude slow waves during NREM sleep and the subtypes of REM sleep were shown to contribute to a better separation between physiological and pathological HFOs. This study investigated rates and spatial spread of the different HFO types (physiological and pathological ripples in the 80–250 Hz frequency band, and fast ripples above 250 Hz) depending on time spent in sleep across the different sleep cycles.

**Methods:**

Fifteen patients with focal pharmaco-resistant epilepsy underwent one night of video-polysomnography during chronic intracranial EEG recording for presurgical epilepsy evaluation. The HFO rate and spread across the different sleep cycles were determined with an automatic HFO detector. We built models to explain the observed rate and spread based on time in sleep and other variables i.e. sleep stage, delta band and sigma band activity, and slow wave amplitude. Statistical significance of the different variables was determined by a model comparison using the Akaike information criterion.

**Results:**

The rate of HFOs depends significantly on the accumulated time of sleep. As the night advanced, the rate of pathological ripples and fast ripples decreased during NREM sleep (up to 15% per hour spent in the respective sleep stages), while the rate of physiological ripples increased during REM sleep (8% per hour spent in REM sleep). Interestingly, the stage of sleep but not the sleep cycle determined the extent of spread of HFOs, showing a larger field during NREM sleep and a more restricted field during REM sleep.

**Conclusion:**

The different dependence with sleep time for physiological and pathological ripples is in keeping with their distinct underlying generating mechanisms. From a practical point of view, the first sleep cycle seems to be best suitable for studying HFOs in epilepsy, given that the contrast between physiological and pathological ripple rates is largest during this time.

## Highlights

• The rate of the different HFO types depends on the duration of sleep.
• The rate of pathological HFOs decreases during NREM sleep as the night advances.
• The rate of physiological HFOs increases during REM sleep as the night advances.
• The stage of sleep determines the extent of spread of the different HFO types.
• Given the largest contrast, HFOs are best studied during the first sleep cycle.

## 1 Introduction

High-frequency oscillations > 80 Hz (HFOs), which can be divided into ripples (80–250 Hz) and fast ripples (> 250 Hz), are a new biomarker of epilepsy (see review of Frauscher et al., 2017, submitted). Of note, ripples and even fast ripples have been described to occur also in normal cortical areas, such as the paracentral cortex, the hippocampus, and the occipital cortex (Axmacher et al., 2008, Blanco et al., 2011, Nagasawa et al., 2012, Melani et al., 2013, Alkawadri et al., 2014, von Ellenrieder et al., 2016, Nonoda et al., 2016). Traditional markers (ripple rate per minute, power, duration, and amplitude) as well as the relation to epileptic activity, presence of task-induced HFOs, or oscillatory EEG background activity are unable to successfully separate physiological from pathological HFOs (Nagasawa et al., 2012, Matsumoto et al., 2013, Melani et al., 2013, Wang et al., 2013, Kerber et al., 2014, Alkawadri et al., 2014, Malinowska et al., 2015).

The stage of sleep modulates the occurrence of HFOs. They have highest rates during NREM sleep, and lowest rates during REM sleep (Staba et al., 2004, Bagshaw et al., 2009, Dümpelmann et al., 2015, Sakuraba et al., 2016). Sleep can separate physiological from pathological HFOs (Frauscher et al., 2015, Frauscher et al., 2016, von Ellenrieder et al., 2016, Nonoda et al., 2016). For instance, HFOs occurring in normal cortical areas (physiological HFOs) are coupled to a different phase of the high amplitude slow wave compared to HFOs occurring in the epileptogenic zone (pathological HFOs) (Frauscher et al., 2015). Also, there is a difference in the coupling of physiological and pathological HFOs to the two subtypes of REM sleep: Pathological HFOs are maximally suppressed during phasic REM sleep compared to tonic REM sleep, whereas physiological HFOs show the opposite behavior with higher rates during phasic compared to tonic REM sleep (Frauscher et al., 2016). These studies suggest that the coupling to sleep transients might be useful to separate physiological from pathological HFOs. For instance, adding the coupling to slow waves increases the discrimination between physiological and pathological HFOs (von Ellenrieder et al., 2016).

Visual HFO identification is traditionally performed in five-minute segments of NREM sleep (Zelmann et al., 2009). It is currently not known during which sleep cycle these segments should be best selected, as the distribution of HFOs depending on time in sleep across the night has not been investigated so far. Given the dependence of HFO rates with the stage of sleep, slow wave amplitude, delta band activity, sleep spindles, and type of REM sleep (Frauscher et al., 2017 submitted), we speculated that rates of the different HFO types might also change across the different sleep cycles throughout the night, which is not solely explained by the stage of sleep.

It is also unknown if the different sleep stages and cycles influence or modulate the spatial spread, or field, of HFOs. Based on findings of interictal epileptic discharges (IEDs), which were shown to be more widespread during NREM sleep and more focally restricted during REM sleep (Sammaritano et al., 1991), it is tempting to speculate that HFOs might also have a wider field during NREM sleep as opposed to REM sleep. This study analyzed if rates and spatial spread of physiological and pathological ripples, and fast ripples depend on the time in sleep and vary across the sleep cycles, beyond the dependence with the stages of sleep.

## 2 Material & methods

### 2.1 Patient selection

We selected patients with pharmaco-resistant focal epilepsy who underwent combined scalp-intracerebral EEG recording (S-EEG electrodes) for presurgical epilepsy evaluation at the Montreal Neurological Institute and Hospital between October 2013 and January 2015, and one night of video-polysomnography during the S-EEG investigation. We included patient recordings which had at least one channel in the physiological region and one channel in the pathological region (see definitions below), as we aimed to evaluate both physiological and pathological HFOs. Exclusion criteria were: (i) scalp EEGs with IEDs (spikes, sharp waves, or polyspike waves with or without after discharge slow wave) or widespread pathologic slowing during wakefulness making correct sleep staging ambiguous or impossible; and (ii) presence of secondarily generalized seizures during the 12 h, or focal seizures (symptomatic or asymptomatic, habitual or non-habitual) during the 6 h prior to or during the evaluated night of sleep recording.

Thirty patients underwent intracerebral EEG with at least one night of video-polysomnography, and 15 were included in the current project according to the selection criteria. Reasons for exclusion were occurrence of focal seizures during the 6 h prior to or during the evaluated night of sleep recording (n = 6), absence of normal EEG channels (n = 6), and scalp EEGs making sleep staging ambiguous or impossible (n = 3). Table S1 of the Supplementary File A provides information on the demographic, neuroimaging, and electroclinical findings of the patient group. This study was approved by the Montreal Neurological Institute and Hospital Review Ethics Board. All patients signed an ethical board approved written informed consent prior to study participation.

### 2.2 Scalp and intracerebral EEG recordings

Intracerebral EEG electrodes were implanted stereotactically using an image-guided system. Table S1 of the Supplementary File A provides the investigated cortical sites. Scalp EEG was obtained with subdermal thin wire electrodes at positions F3, F4, Fz, C3, C4, Cz, P3, P4, and Pz. In the night of the sleep recording, which was at least 72 h after implantation, additional electrodes for electrooculography and electromyography of the chin and the flexor digitorum superficialis muscles were used. The EEG signal was high-pass-filtered at 0.1 Hz, low-pass-filtered at 500 Hz, and sampled at 2000 Hz. EEG were recorded using the Harmonie EEG system (Stellate, Montreal, Canada). Sleep was scored manually in 30 s epochs in the scalp EEG by a sleep expert (Berry et al., 2012).

Intracerebral EEG channels were classified as channels in the physiological region or channels in the pathological region. Channels in the physiological region had normal EEG activity (absence of IEDs and of non-epileptic abnormalities during the complete intracranial recording, usually lasting 2–3 weeks), were located in brain regions with no structural abnormalities as revealed by high-resolution MRI, and were outside the seizure-onset zone (i.e. showing the first unequivocal ictal intracranial EEG change at seizure onset of both habitual and non-habitual seizures, see Spanedda et al., 1997). Channels in the pathological region included channels inside the irritative zone (i.e. with IEDs) and channels in the seizure-onset zone. Channels displaying non-epileptic abnormalities, artifacts interfering with the identification of HFOs, or channels outside the brain were excluded. Suitable channels were selected independently by two electrophysiologists.

### 2.3 HFO detection

HFOs were automatically detected looking for an increase in power with respect to the background in narrow frequency bands and with a duration longer than four oscillations plus the effective response time of the filters (equi-ripple FIR filters of order 508, more details in von Ellenrieder et al., 2012, von Ellenrieder et al., 2016). Ideally, a human reviewer should verify the results of an automatic detector, but in this case, such an approach was not practical, since the whole night was investigated. For this reason, and since muscle activity and movement artifacts could lead to an increase of false positives in the HFO detection, we excluded wake and stage N1 sleep from the analysis.

We studied the subject-level *rate* of HFOs, defined by the number of occasions in which an HFO is detected in one channel or several channels simultaneously, i.e. when HFOs are detected simultaneously in several channels, it counts as one subject level event. We also studied the HFO spatial *spread*, defined as the number of channels in which HFOs are detected during each subject-level event. When studying the spread of HFOs, we excluded all the subject-level events that involved channels in the physiological and pathological regions, since in such cases it was not possible to determine if the ripple was pathological or physiological.

### 2.4 Variables included in the model

The primary variable of interest is the time spent in sleep in any given sleep stage. Other variables that could lead to HFO rate and spread changes were included in the model as well. These secondary variables are the respective sleep stages (REM, N2, and N3), the slow wave amplitude, the delta band activity, and the sigma band activity (10–16 Hz). The slow wave amplitude is the average amplitude of the slow waves detected in 30 s epochs used in the sleep scoring, defining slow waves as oscillations of the band pass filtered signal (0.5–4 Hz) with consecutive zero crossings separated by 0.5 to 2 s. See von Ellenrieder et al. (2016) for more details on the filters and slow wave detection algorithm. The delta band and sigma band activity was computed as the root mean square value of the band pass filtered signal during the same 30 s epochs (elliptic IIR filters of order 5, 0.2 dB ripple in the pass band 40 dB attenuation in the stop bands, 0.5–4 Hz and 10–16 Hz respectively). All the variables were computed for the scalp channels F3-C3 and F4-C4 and averaged, then modified to have zero mean in each analyzed sleep stage and/or patient, and all the variables except the accumulated time were normalized to have unit variance in each patient. The accumulated time was expressed in hours.

### 2.5 Models

In order to study the variation of the rate and spread of HFOs, we fitted the measurements to mathematical models. The HFO rate is modeled as a Poisson process, in which no overlap between events is allowed, and the time intervals between consecutive events are statistically independent. This model is described by a single parameter, either the mean duration of the intervals between events or its inverse, the mean rate. The proposed models attempt to estimate the mean rate for every 30 s interval coincident with the sleep staging of the 30-s epochs, as a function of the variables described above.

We expressed the time-varying rates as the mean rate computed over the whole investigated period, and relative variations of this mean rate depending on all studied variables (sleep stage, accumulated sleep time, slow wave amplitude, delta, and sigma band activity). We considered the mean rate as patient-specific, since it depends on the particular pathology (Ferrari-Marinho et al., 2016) and implantation scheme of the patient (i.e. number of electrode contacts in pathological as opposed to physiological regions).

On the other hand, for the rate variations in time we were interested in common features of the studied patients. Hence, in our model the relative variations around the mean are the same across subjects (see Supplementary File B for the full mathematical expressions for the models).

To study the variation of the spatial spread of HFOs we approximated it by a geometric model, in which the probability of involving one extra channel is independent of the number of channels already involved. This model is also completely described by a single parameter, e.g. by the mean. Thus, the same decomposition described for the mean rate was used to model the mean spread (see Supplementary File B).

### 2.6 Statistical hypothesis testing

We performed the statistical hypothesis testing with a model comparison approach using the Akaike Information Criterion (AIC) (Burnham and Anderson, 2002). The AIC value of a model takes into accounts the goodness-of-fit of the model, and its complexity. When comparing models that differ in only one variable, the AIC value difference indicates whether the particular variable has a statistically significant contribution to the model. The AIC reduces to an F-test, when comparing nested Gaussian models, but it can be applied to compare also non-nested non-Gaussian models (see Supplementary File B for the computation of the AIC value for the proposed models).

## 3 Results

In the 15 patients, a total of 9,211 30 s epochs were analyzed, 2211 scored as REM, 4724 as N2, and 2276 as N3 sleep. We studied HFOs by analyzing the number of events at subject level. Almost ninety thousand (87,783) subject-level events involving only channels devoid of epileptic activity were detected in the ripple band (80–250 Hz), and referred as *physiological ripples*. Events in this frequency band, but involving only channels in the irritative zone or in the seizure-onset zone are referred as *pathologic ripples*; this category was the most numerous group (303,175). Finally, 35,111 *fast ripples* were detected in the 250–500 Hz band involving at least one channel in the irritative zone or seizure-onset zone. The fast ripples detected only in channels in the physiological region (a total of 5605 fast ripples, average one every 1.15 min) were not included in the analysis. More than half of these fast ripples were detected in the occipital cortex of a single patient; such fast activity has been reported in the normal occipital cortex before (Blanco et al., 2011, Nagasawa et al., 2012, Melani et al., 2013, Nonoda et al., 2016). The remaining fast ripples are probably false detections of the automatic detector.

### 3.1 Average subject-level rates

**Fig. 1:**
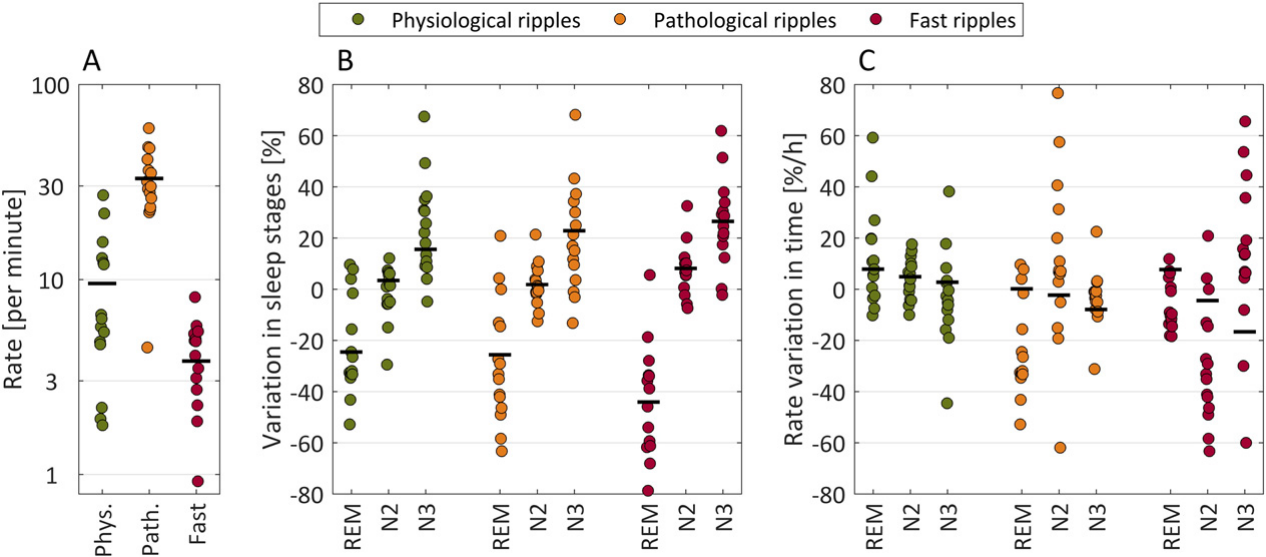
A. Average of the subject-level HFO rate of each patient throughout the night. Each dot corresponds to the value of a single subject and the black lines represent the mean value for all patients. In general the rate of pathological ripples is higher than the rate of physiological ripples and fast ripples. However, there are large variations, since the absolute value of the rate depends on the implantation scheme and pathology of the patients. B. Effect of sleep stages. Relative variation of the rate in REM, N2, and N3 sleep stages with respect to the individual averages shown in A. This panel confirms results that have been previously reported in the literature, showing that HFO rates are higher during NREM compared to REM sleep. C. Effect of accumulated time spent in each sleep stage. The panel shows the relative change in rate per hour spent in any of the sleep stages. The graph shows that, as time passes, the rate of physiological ripples and fast ripples increases, when patients are in REM sleep (positive rate variation). In contrast, the rate of pathological ripples and fast ripples decreases, when patients are in NREM sleep, particularly in N3 (negative rate variation).

The average subject-level HFO rates are presented in Fig. 1A, which shows that in general the highest rates are observed for pathologic ripples, followed by physiologic ripples, and fast ripples. However, there is a large variability of these rates across the different patients. Note that the subject-level rate depends in each patient on the number of channels recording from the irritative zone or seizure-onset zone and outside of them, in addition to pathology. We do not analyze further these average subject-level rates, but focus on the relative variation of the rates due to accumulated sleep time, sleep stage, slow wave amplitude, delta band activity, and sigma band activity, which should not be strongly affected by the number of channels recorded in each patient.

### 3.2 Sleep stages

Allowing a different average rate for each sleep stage leads to a significantly better model than any model that does not include it, as can be seen in Table S2 of the Supplementary File A. The first row in Table 1 shows the coefficients for each stage and HFO type. Across all types, there is a large increase in the HFO rate during N3 sleep (16.7% for physiological ripples, 23.7% for pathological ripples, and 27.1% for fast ripples) and an important decrease during REM sleep (− 23.6% for physiological ripples, − 25.2% for pathological ripples, and − 44.0% for fast ripples). These coefficients correspond to the whole cohort, showing variations that are highly significant at a group level (see Fig. 1B).

**Table 1:** Coefficients of the relative rate variation for the best model.

	Physiological ripples			Pathological ripples			Fast ripples		
	REM	N2	N3	REM	N2	N3	REM	N2	N3
Stage	− 23.6*	4.6*	16.7*	− 25.2*	2.3*	23.7*	− 44.0*	8.5*	27.1*
AT	7.7*	7.0*	3.3*	2.8*	− 0.7*	− 7.7*	11.5*	− 0.6	− 14.3*
SW	− 0.9	1.8*	4.5*	3.7*	4.9*	8.4*	3.2*	6.0*	5.7*
DA	–	–	–	− 4.0*	− 1.6*	− 3.0*	− 7.1*	− 4.5*	− 3.0*
SA	0.5	4.4*	− 1.8*	7.4*	1.7*	− 2.8*	16.2*	9.4*	1.1

Units are percentages, except in the case of accumulated time, with units of percentage per hour. Asterisks indicate the coefficients significantly different from zero. For example, there is a 3.3% increase per hour during N3 sleep for physiological ripples, and a 14.3% decrease per hour for fast ripples. Legend. AT: accumulated time; SW: slow wave amplitude; DA: delta band activity; SA: sigma band activity.

### 3.3 Accumulated time in sleep

**Fig. 2:**
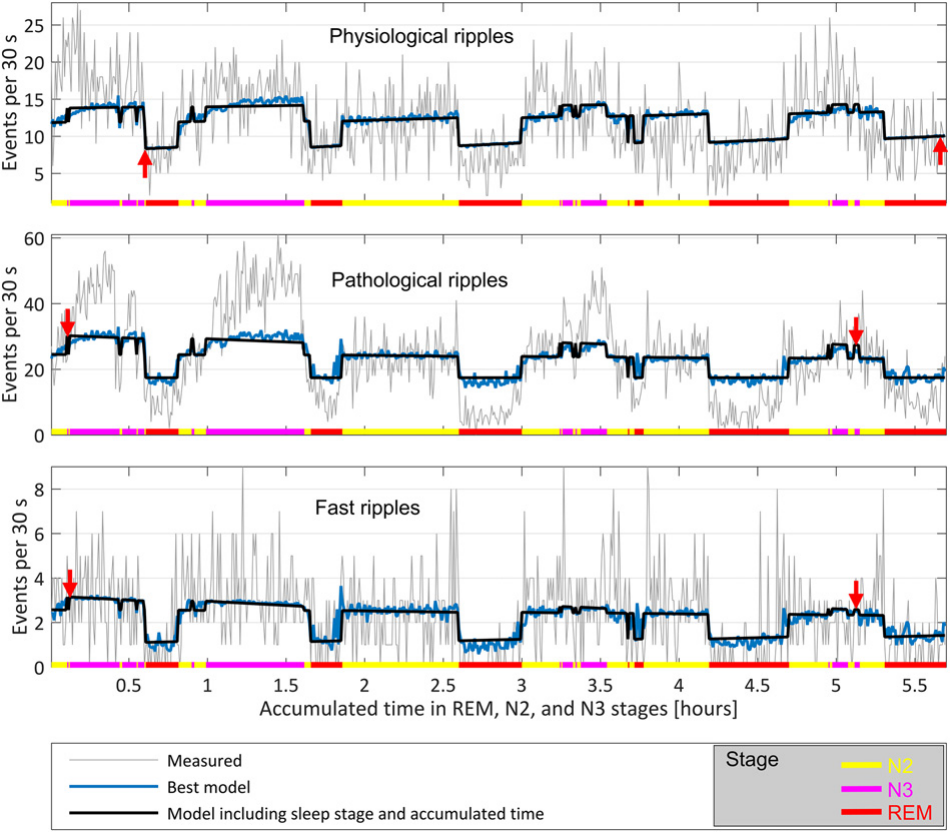
Example showing the HFO rate and the time varying rate predicted by two Poisson process models common for every subject. The prediction in black corresponds to a model taking into account sleep stage and accumulated time in each stage. The prediction of the best model is shown in blue, and includes also the variables measuring the slow wave amplitude, the sigma band activity, and in the case of pathological ripples and fast ripples also the delta band activity. The rate is plotted as a function of time, and the sleep stage is indicated in color at the bottom of the graphs. Observe the rate increase with time during REM sleep for physiological ripples by comparing the vertical position of the red arrows, and a decrease during NREM sleep for pathologic ripples and fast ripples (most marked during stage N3, indicated also by the difference in the vertical position of the arrows). Note that the rate of physiological ripples is always below 15 per hour across the whole night's NREM sleep, and the rate of pathological HFOs is above 20 per hour during NREM sleep, but there is a larger difference in physiological and pathological HFO rates during the first sleep cycle as compared to the last sleep cycle.

Including the accumulated time always leads to an improvement of the models (Table S2 of the Supplementary File A). The effect of accumulated time in each of the sleep stages and HFO types is shown in Fig. 1C and AT row of Table S3 in the Supplementary File A. As the night progresses, the HFO rates increase on average during REM sleep (physiological ripples at a rate of 7.8% per hour, pathological ripples 0.1% per hour, and fast ripples 7.7% per hour). During non-REM sleep, the physiological ripple rate increases with time (4.8% per hour in N2, 2.7% per hour in N3), and the pathological ripple and fast ripple rates decrease with time (− 2.3% per hour in N2 and − 7.9% per hour in N3 for pathological ripples, − 4.5% per hour in N2 and − 16.7% per hour in N3 for fast ripples). These coefficients correspond to the whole cohort, showing significant variations at the group level. Fig. 2 shows a representative hypnogram of one patient illustrating this behavior.

### 3.4 Low frequency activity

The delta band activity and the slow wave amplitude are positively correlated with the HFO rates during NREM sleep (Supplementary Table S3, rows SW and DA, columns N2 and N3). During REM sleep there is less low frequency activity and the practical significance of these variables is not clear. The delta band activity and slow wave amplitude are highly correlated variables, especially during NREM sleep (correlation coefficient 0.81 during N3, 0.72 during N2, and 0.55 during REM), and, when including both variables in the model, the coefficients change compared to the behavior described for the individual variables. In NREM sleep the coefficient describing the dependence with delta band activity takes positive values, and the one associated to slow wave amplitude takes negative values (Supplementary Table S3, row SW DA, columns N2 and N3). In REM sleep, the behavior is less consistent, probably due to the absence of low frequency activity.

### 3.5 Sigma band activity

The sigma band activity is positively correlated to the rate of all types of HFOs during N2, and negatively correlated to physiological and pathological ripple rates during N3. For fast ripples the correlation is positive in N3, but of much smaller rate compared to N2 sleep. This might point to an association between HFOs and spindles, which are more prevalent during N2 sleep. This association is similar to the one with IEDs, as previously shown (Ferrillo et al., 2000).

During REM sleep the sigma band activity shows a strong positive correlation to pathological ripple and fast ripple rates, while there is no evidence of a correlation to physiological ripple rates. It should be noted that this result does not necessarily point out to rhythmic sigma activity during REM sleep; it could also be explained by the presence of a few IEDs, contributing to a slight increase in the sigma band power being at the same time accompanied by a higher likelihood of an occurrence of pathological HFOs.

### 3.6 Best model

The coefficients of the best model for each type of HFO can be seen in Table 1. The model incorporating all the variables, i.e. the sleep stage, accumulated time in the stage, delta and sigma band activity, and slow wave amplitude is the best model in the case of pathological ripples and fast ripples. In the case of physiological ripples the best model does not include the delta band activity, but it is not significantly better than the model including all the variables (Supplementary Table S2).

The correlation between pairs of variables other than the slow wave amplitude and delta band activity is not very high (highest absolute correlation coefficient − 0.40 in N3 between sigma band activity and accumulated time). As a result, the coefficients do not change much compared to the cases with a single variable, as shown in Supplementary Table S3 (compare e.g. row AT, SW + DA, SA to row AT + SW + DA + SA). In summary, all types of HFO are more abundant during N3 sleep, and less during REM sleep. Pathological ripple rates and fast ripple rates are positively correlated with accumulated time during REM sleep, and negatively correlated during NREM sleep. They are positively correlated to the slow wave amplitude and negatively correlated to the delta band activity, and positively correlated to the sigma band activity during REM. In contrast, physiological ripples are positively correlated with accumulated time in all sleep stages, i.e. they increase through the night in every sleep stage, and are positively correlated to the slow wave amplitude during NREM sleep, but uncorrelated during REM sleep, and not correlated in addition to the delta band activity. They are positively correlated to the sigma band activity during N2 sleep, negatively correlated during N3 sleep, and uncorrelated during REM sleep.

### 3.7 Spread

**Fig. 3:**
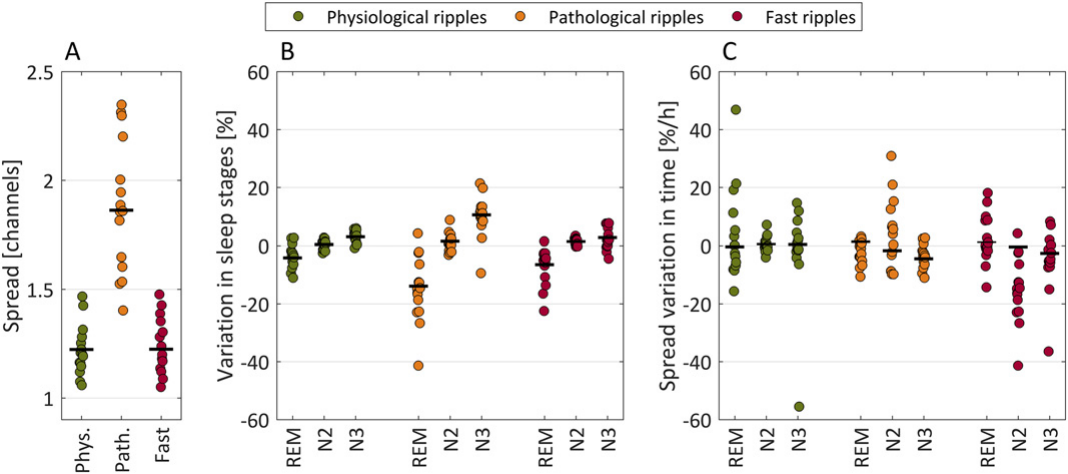
A. Average of the subject level HFO spread of each patient throughout the night. Each dot corresponds to the value of a single subject and the black lines represent the mean value for all patients. In general the spread of pathological ripples is higher than the spread of physiological ripples and fast ripples. However, there are large variations, since the absolute value of the spread depends on the implantation scheme and pathology of the patients. B. Effect of sleep stages. Relative variation of the spread in REM, N2, and N3 sleep stages with respect to the individual averages shown in A. The graph shows that the spread is lower in REM compared to non-REM sleep. C. Effect of accumulated time spent in each sleep stage. The panel shows the relative change in spread per hour spent in any of the sleep stages. The graph shows a slight decrease on average of the spread of pathologic ripples and fast ripples when patients are in NREM sleep. Overall, the relative differences with respect to the average are lower for the spread than for the rate, as seen when comparing this figure to Fig. 1.

The average subject-level spreads are presented in Fig. 3A, which shows that in general the highest spread is observed for pathologic ripples. Six percent of the ripples were excluded from the spread analysis because they involved channels in both physiological and pathological regions.

The quality of the model used to approximate the HFO spread can be assessed in Fig. S1 of the Supplementary File A, which shows the histogram and fitted distributions. The pathological ripples have an average spread of about 2 channels, indicating that it is not uncommon to find them in two or three channels simultaneously. For physiological ripples and fast ripples the average spread is between 1.2 and 1.3 channels, indicating that they are usually detected in just one channel.

**Fig. 4:**
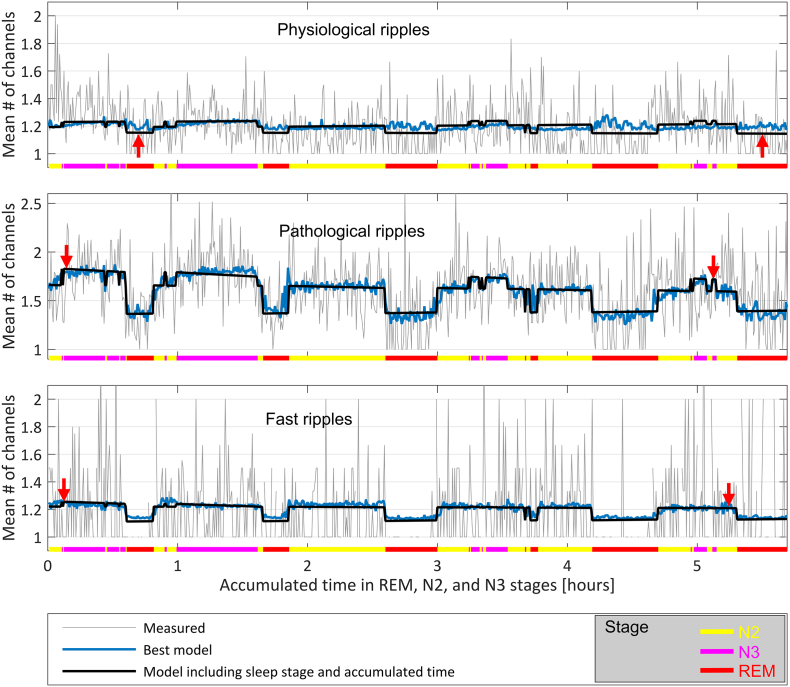
Example showing the measured HFO spread and the time varying spread predicted by two Geometric models common for every subject. The prediction in black corresponds to a model taking into account sleep stage and accumulated time in each stage. The prediction of the best model is shown in blue, and is based only on the delta band activity for physiological ripples, on the sleep stage, sigma and delta band activity and slow wave amplitude for pathological ripples, and on the slow wave amplitude and sigma band activity for fast ripples. The spread is plotted as a function of time, and the sleep stage is indicated in color at the bottom of the graphs. Observe that the spread remains almost constant in time by comparing the vertical position of the red arrows.

The variation of the spread across the different sleep stages can be observed in Fig. 3B, and the variation in time in Fig. 3C. The variations are in the same direction as observed in Fig. 2 for the HFO rates, but of lower magnitude not reaching statistical significance (see Supplementary Tables S3 and S5 for a comparison of the models). The best models are given in Table 2, and they do not include the accumulated sleep time. For the physiological ripple spread the best model does not discriminate between sleep stages and has a dependency only on the delta band activity. For the pathological ripple spread the best model discriminates among sleep stages and depends also on the slow wave amplitude, delta and sigma band activity. For the fast ripple spread the best model does not discriminate sleep stages and only depends on the slow wave amplitude and the sigma band activity. However, these models are not significantly better than many others (see Table S4 of the Supplementary File A). It is important to note that when models do not depend directly on the sleep stage, they can still incorporate stage specific variations through variables that depend on the EEG activity, i.e. SW, DA, and SA. In fact, if these variables are excluded, the models are significantly better when they include the sleep stage (first row of Supplementary Table S4). Fig. 4 shows a representative example of the variation of the spread throughout the night for one patient.

**Table 2:** Coefficients of the relative spread variation for the best models.

	Physiological ripples	Pathological ripples			Fast ripples
	All stages	REM	N2	N3	All stages
Stage	–	− 13.9*	1.4*	10.5*	–
AT	–	–	–	–	–
SW	–	2.0*	2.0*	3.5*	0.7*
DA	1.8*	− 2.8*	− 0.5	− 0.8	–
SA	–	4.0*	2.8*	1.5	3.4*

Units are percentages. Asterisks indicate the coefficients that are significantly different from zero. For example, there is a 13.9% decrease in spatial spread of pathological ripples during REM sleep, as opposed to a 10.5% increase during N3 sleep. Legend. AT: accumulated time; SW: slow wave amplitude; DA: delta band activity; SA: sigma band activity.

## 4 Discussion

This study investigated the time in sleep-dependent properties of rate and spatial spread of the different types of HFOs. The major findings of this work are (i) the presence of a sleep-homeostatic variation of the rate of HFOs across the different sleep cycles, (ii) a difference in the behavior of physiological versus pathological HFOs with sleep time, with the highest difference in rates of physiological and pathological HFOs occurring during the first sleep cycle, and (iii) a modulation of the spread of HFOs by the different sleep stages, but not the different sleep cycles.

### 4.1 Dependence of the rate of HFOs on sleep duration

This study demonstrates that the rate of HFOs does not only depend on the different sleep stages, but also on the total duration of sleep. Of note, this dependence cannot be solely explained by previously reported sleep-homeostatic changes in delta power or slow wave amplitude across the night (Riedner et al., 2007). This is important, as both features were independently shown to be correlated with the rate of HFOs (Nagasawa et al., 2012, Frauscher et al., 2015, von Ellenrieder et al., 2016, Nonoda et al., 2016).

### 4.2 Different behavior of physiological and pathological HFOs across the night

Interestingly, the physiological ripple rate, those in channels devoid of epileptic activity, increased with time during REM sleep. In contrast, in channels with epileptic activity, the ripple and fast ripple rates decreased with time during NREM sleep. We believe that this is an important observation that should help differentiating physiological and pathological ripples.

The behavior of HFO types with respect to sleep homeostasis likely reflects the different underlying mechanisms of these physiological and pathological oscillations. The physiological ripples are thought to reflect summed excitatory postsynaptic potentials, while pathological ripples and fast ripples reflect summed action potentials of synchronously bursting neurons (Engel et al., 2009, Jefferys et al., 2012).

Our data suggest that pathological HFOs follow a distribution pattern similar to the sleep-homeostatic variations of slow waves (Riedner et al., 2007), whereas physiological HFO rates increase across the night. The behavior of pathological HFOs further underlines that synchronization, which is most marked during high-amplitude slow waves, is important for their generation. This decrease with time of sleep is more pronounced for fast ripples than for pathological ripples. This difference, however, can be explained by the fact that the ripples defined as pathological in this study, might represent a mixed population of true epileptic ripples and of physiological ripples also present in the pathological region.

In contrast, the increase in physiological HFOs across REM sleep might be due to the known increase of the amount of phasic REM sleep in later sleep cycles (Peters et al., 2014). In a previous work, we showed that physiological HFOs are closely linked to phasic REM sleep (Frauscher et al., 2016), which is suggested to play an important role in learning and memory (Buzsáki et al., 1992, Datta, 2000, Datta et al., 2004, Diekelmann et al., 2009).

The different behavior between both ripple types is larger at the beginning of the night. This finding is not only interesting from a pathophysiological consideration, but is also of practical relevance, as our data suggest that the detection of HFOs in epilepsy is recommended to be performed during the first sleep cycle.

### 4.3 The stage of sleep, but not the sleep cycle determines the extent of the spread of HFOs

We found that the stage of sleep, but not the sleep cycle determines the extent of spread of HFOs. This third important finding demonstrates that the stage of sleep does not only influence the rate of HFOs, but also their spread. The rate of HFOs is higher with a larger field during NREM sleep as opposed to lower rates of a more focal distribution during REM sleep. This finding further supports the notion that epileptic activity during REM sleep is more specific for the SOZ compared to NREM sleep (Sammaritano et al., 1991). Sakuraba et al. (2016) recently demonstrated that HFOs near the epileptogenic zone are less suppressed during REM sleep, and are a particular useful marker for identification of the SOZ.

### 4.4 This study confirms the dependence of HFO rates on the different sleep stages

Finally our results confirmed the role of the different stages of sleep on HFO rates (Staba et al., 2004, Bagshaw et al., 2009, Dümpelmann et al., 2015, Sakuraba et al., 2016). Their rates are highest during NREM sleep, whereas lowest rates are observed during REM sleep. Moreover, we replicated here the known relationship with delta band activity, and with the amplitude of the sleep slow waves (Nagasawa et al., 2012, Frauscher et al., 2015, Nonoda et al., 2016).

### 4.5 Limitations

Given the large amount of data, we used an automatic HFO detector (von Ellenrieder et al., 2012, von Ellenrieder et al., 2016), but automatic detection may lead to detection of false positive events. As the rate of false positives would be particularly high during wakefulness and N1 sleep due to movement and muscle artifacts, we excluded these stages from analysis. The distribution pattern of HFOs across the different sleep stages is in line with the existing literature (Staba et al., 2004, Bagshaw et al., 2009, Dümpelmann et al., 2015, Sakuraba et al., 2016) corroborating the validity of the automatic HFO detection in this work. We cannot completely rule out that the heterogeneity of the studied patients and the use of antiepileptic medication, known to have potentially altering effects on sleep (Jain and Glauser, 2014), might have had an influence. The fact that our findings reached significance across all patients irrespective of the type of epilepsy and antiepileptic medication, does underline the robustness of the effect of sleep duration on the rates and spread of HFOs. The set-up of intracranial EEG recording in the epilepsy monitoring unit might have resulted in a higher degree of sleep fragmentation compared to the patients' home environment. In order to minimize this influence, we selected to perform the sleep recording a minimum of 72 h after the electrode implantation, a time where patients had usually adapted to the environment of the epilepsy monitoring unit and where effects from anesthesia and headaches due to electrode placement are remitted.

## 5 Conclusion

This study demonstrated that HFO rates do not only depend on the sleep stages, but have also a significant sleep-homeostatic variation across the different sleep cycles. Moreover, we found a difference in the behavior of physiological versus pathological HFOs with sleep time, with the highest difference in rates of both HFO types occurring during the first sleep cycle. This is best explained by their different underlying generating mechanisms. From a practical point of view, the first sleep cycle seems therefore to be best suitable for studying pathological HFOs in epilepsy.

## Disclosures of conflict of interest

No financial disclosures related to this project have to be disclosed. Outside of the submitted work, B.F. has received a speaker's fee from Novartis Japan, and advisory board and speaker's honoraria, as well as congress travel support from UCB Pharma. N.v.E. and J.G. have received fees for consultancy from Precisis Inc. F.D. has nothing to disclose.

## Appendix A Supplementary data

Supplementary Tables and Figures. Image 1

Mathematical and statistical models. Image 2
