# Supplementary material for: Physiological and pathological high-frequency oscillations have distinct sleep-homeostatic properties
Source: Neuroimage Clin. 2017 Feb 24;14:566–73. doi: 10.1016/j.nicl.2017.02.018 (PMC5349616; doi:10.1016/j.nicl.2017.02.018)
Supplement: Supplementary file 1 — Supplementary Tables and Figures. [file mmc1.pdf]

SUPPLEMENTARY MATERIAL

SUPPLEMENTARY TABLES

Supplementary Table S1. Demographic, neuroimaging, and electrophysiological data of the patient cohort

ID	Ictal and Interictal Scalp EEG Findings	MR imaging	Implanted electrode positions	Interictal SEEG	SOZ SEEG	Surgery following SEEG	Pathology	Antiepileptic Medication at time of Investigation
1	LT	no lesion	L: OF,Ca, Cm,Is,Ip, Am,Hc,Hp,Tp,P Hp<br>R: Hc	L: Am, Hc, Hp, Tp and T neocortex, Is<br>R: Hc	L T and L perisplenial area > L I >>> L T mesial	L ant T and Am	unspecified gliosis	TPX (200)<br>CBZ (600)<br>Clo (30)
2	bil T, L>R	bil focal PNH: RF, R + L atrium, LO horn with L TPO abnormal gyration and atrophy	L: OF, Ca, Am,Hc,Hp,Fus,C p,PC,Li<br>R: Am,Hc	R: Am<br>L: Am, Hc, Hp, Fus, Li, OF, CP, PC	R Am, L Fus and L T neocortex	L O horn NH thermocoagulation	---	LEV (3000)<br>Clo (25)
3	L FT	no lesion	L: OF,Ca, Ia,Am,Hc,Hp,Ec, Fus,Ci	L: Hc + ant T neocortex, Am, Ia	L ant insula and ant T neocortex	No	---	CBZ (1200)
4	L TPO	L post insula, T and inf P atrophy and gliosis	L: OF,Ia, Ip,Hp,Ci, Pc,Cu,Os	L: Hp,Pc,Cu,Os	L O and Hp	L O	FCD type III	LEV (3000)<br>PHT (350)<br>Clo (40)
5	bil T	bil focal PNH: L + R atrium, L Fus, L + R T, L O horn	R: Ca,Hc, Hp<br>L: OF,Ca, Am,Hc,Hp, Fus,Cp	R: F NH, T NH<br>L: F NH, Am, Hc + neocortex, T NH, Fus NH + overlying	L T or bilateral T L > R, 1x R T mesial	L ant T	glial neuronal heterotopia	VPA (2250)<br>LEV (3000)<br>CBZ (1400)

				neocortex				
6	bil T	L Hc atrophy	L: Am,Hc, Hp,Fus<br>R: Am,Hc, Hp,Fus	L: Am,Hc, T neocortex<br>R: Am, Hc, T neocortex	T neocortex, R>L	no	---	OXC (1800)<br>LEV (3000)<br>LTG (400)
7	bil FT	R Hc atrophy	L: OF,Ca, Cm,Am, Hc,Hp<br>R: OF,Ca, Cm,Am,Hc,Hp	R: T, OF<br>L: T, neocortex and Am	R: Hp, R T neocortex<br>L: T neocortex, Am, Hc, Hp	R SeAH	Hcsclerosis	VPA (2000)<br>Clo (30)<br>LEV (3000)
8	L T	L Hc atrophy	L: Ca,Am, Hc,Hp,Fus, Ci,Oi	L: Am, Hc, Hp, Fus + T neocortex	L Hc and Fus	L SeAH	Hc sclerosis	LTG (400)<br>CBZ (800)
9	R TPO	no lesion	R: Am,Hc, Hp,Ci,Cis, PC,Os,Oi	R: Am, Hc, Hp, Ci	R Hc and inf isthmus	R SeAH	Hc gliosis	LEV (2000)
10	L TPO	no lesion	L: Am,Hc, Hp,Fus,Ci,LL<br>R: Am,Hc	L: mesial + neocorticalT,Fus ,Ci<br>R: mesial + neocortical T	L T-O (post basal T and Fus, and ant lingual)	no	---	CBZ (1200)<br>LTG (300)<br>Clo (20)
11	bil FT	bil Hc sclerosis	L: OF,Am,Hc,Hp<br>R: OF,Am, Hc, Hp	L: AM, Hc, Hp<br>R: Am, Hc, Hp	bil T mesial	no	---	CBZ (1000)<br>LTG (400)<br>LEV (2500)
12	R FCP	lesion R post insula + transverse gyrus, remote cortical resection R F operculum, R T1, R ant T2	L: Ca,SMA,M,S, SMi,SMs,Cp, Ia,Ip, Hc,Hp,pT,He	L: T neocortex, He, transverse gyrus, Ip	L He, transverse gyrusand Ip	L transverse gyrus + Heschl	unspecified gliosis	CBZ (1800)<br>LTG (400)<br>Clo (40)
13	bil T	bil Hc atrophy, remote L SeAH	L: OF,Am,Hp,Ia, Ip<br>R: OF,Am,Hc,Hp	L: residual T mesial structures<br>R: T mesial	R T mesial	no	---	LAC (300)<br>CBZ (1200)<br>Clo (20)

				>neocortical				
14	FT R >> L T	remote ischemic lesion R thalamus	L: OF,Ca,Cm, Am,Hc, R: Fa,OF,Ca,Cm, Am,Hc	L: Am, Hc R: Hc	Generator not identified	no	---	LTG (400) VPA (1000)
15	R FCT	R T lobe atrophy, signal abnormality residual R Hc, remote R ant T lobe resection	R: OF,Ca, Cm,I,He,Hc,Hp	R: insula, T neocortex, Hc, He	R insula	R posterior T neocortex and residual R Hc	R T lobe: unspecified gliosis; R residual Hc: normal	LTG (200) Clo (20)

Legend. OF, orbitofrontal; Am, amygdala; ant, anterior; bil, bilateral; C, central; Cu, cuneus; Ca, anterior cingulate gyrus; CBZ, carbamazepine; Ci, isthmus of the cingulate gyrus, Cis, superior part of the isthmus of the cingulate gyrus; Clo, clobazam; Cm, middle cingulate gyrus; Ec, entorhinal cortex; F, frontal; Fa, frontal anterior; FCD, focal cortical dysplasia; Fus, fusiform gyrus; Hc, hippocampus; He, Heschl'sgyrus; Hp, posterior portion of the hippocampus; I, insula; Ia, anterior insula; inf, inferior; Ip, posterior insula; Is, superior insula; L, left; Le, lesion; LEV, levetiracetam; LL, lingual gyrus; LTG, lamotrigine; M, primary motor cortex; NH, nodular heterotopia; NHa, anterior to the nodular heterotopia; NHp, posterior to the nodular heterotopia; O, occipital; Oi, inferior occipital; Os, superior occipital; OXC, oxcarabamazepine; P, parietal; PC, Pre-Cuneus; PHp, parahippocampalgyrus; PHT, phenytoine; PNH, periventricular nodular heterotopia; pT, posterior temporal neocortex T1; R, right; S, primary sensory cortex; SeAH, selective amydalo-hippocampectomy; SMisupramarginal inferior; SMs, supramarginal superior; T, temporal; Tp, temporal pole; TPX, topiramate; VPA, valproate acid.

Supplementary Table S2. Akaike Information Criterion (AIC) value, relative to the model with constant rate during the whole investigated period

	Physiological ripples		Pathological ripples		Fast ripples	
	ND	D	ND	D	ND	D
Const.	0	-2032	0	-8689	0	-2594
AT	-32	-2143	-1738	-8849	-403	-2683
SW	-966	-2073	-5985	-9319	-1105	-2679
DA	-524	-2054	-3055	-8918	-481	-2665
SA	-1133	-2082	-5369	-9408	-2419	-3190
AT+SW	-1051	-2214	-6044	-9373	-1137	-2743
AT+DA	-532	-2174	-3577	-9038	-644	-2754
AT+SA	-1179	-2234	-5622	-9575	-2422	-3268
SW+DA	-994	-2068	-6308	-9495	-1230	-2798
SW+SA	-1540	-2114	-7771	-9848	-2586	-3208
DA+SA	-1310	-2096	-6200	-9581	-2428	-3266
AT+SW+DA	-1081	-2209	-6356	-9542	-1257	-2864
AT+SW+SA	-1773	-2280*#	-7769	-9933	-2588	-3279
AT+DA+SA	-1440	-2252	-6280	-9720	-2428	-3355
SW+DA+SA	-1566	-2108	-8079	-9997	-2722	-3321
AT+SW+DA+SA	-1800	-2277*	-8078	-10080*#	-2727	-3395*#

Lower AIC values indicate preferred models. A difference greater than 6 between two models is necessary for statistical significance. Asterisks indicate models that are not significantly different from the best model, indicated by a number sign.

Legend. D, discriminated sleep stages; ND: non-discriminated sleep stages; AT: accumulated time; SW: slow wave amplitude; DA: delta band activity; SA: sigma band activity.

Supplementary Table S3. Coefficients of the relative rate variation for the tested models

	Physiological ripples				Pathological ripples				Fast ripples			
Stage	ND	REM	N2	N3	ND	REM	N2	N3	ND	REM	N2	N3
Variable		-24.6*	3.4*	15.6*		-25.6*	1.7*	22.8*		-44.1*	8.1*	26.5*
AT	-1.1	7.8	4.8	2.7	-4.2	0.1	-2.3	-7.9	-5.9	7.7	-4.5	-16.7
SW	10.8	-1.2	1.2	4.3	14.7	2.8	4.6	6.7	19.1	2.7	6.6	4.2
DA	7.9	-0.3	0.6	3.6	10.4	-1.8	2.6	4.2	12.4	-5.8	2.7	1.6
SA	11.6	0.0	3.0	-3.1	13.8	7.7	3.4	-1.3	27.6	17.0	10.6	3.9
AT	1.9	7.6	5.8	4.5	-0.9	0.4	-1.1	-5.8	-1.9	7.9	-2.7	-15.7
SW	12.4	-0.8	2.7	4.9	14.0	2.8	4.3	6.2	17.5	2.8	5.8	3.2
AT	0.6	7.8	5.1	3.4	-2.4	0.3	-1.9	-7.3	-4.0	8.6	-4.1	-16.5
DA	8.3	-0.1	1.4	3.8	8.8	-1.9	2.3	3.9	9.5	-6.0	1.9	1.3
AT	1.3	7.9	6.5	1.0	-1.7	2.3	-1.5	-10.6	-0.6	10.9	-1.4	-16.1
SA	12.6	0.3	4.7	-2.8	12.6	7.9	3.0	-3.4	27.1	17.2	10.2	0.7
SW	13.6	-1.4	1.4	4.3	19.6	5.0	5.8	9.3	28.3	5.5	10.2	7.7
DA	-3.3	0.4	-0.4	0.0	-5.9	-4.2	-1.7	-3.2	-10.8	-6.1	-4.9	-4.3
SW	7.5	-1.2	0.3	4.0	10.4	1.5	3.8	6.6	8.0	-0.9	2.7	4.5
SA	8.7	0.2	2.9	-2.6	8.8	7.4	1.8	-1.0	23.6	17.2	9.6	4.2
DA	4.7	-0.3	0.0	3.1	5.8	-2.1	1.8	4.1	1.9	-6.4	-0.4	2.0
SA	10.1	0.0	3.0	-2.5	11.4	7.7	2.9	-0.9	26.7	16.9	10.7	4.1
AT	1.9	7.7	5.9	4.5	-0.8	1.0	-1.0	-5.4	-1.7	9.0	-2.4	-15.3
SW	15.2	-1.0	3.3	5.3	18.9	5.0	5.5	8.4	26.6	5.6	9.3	5.6
DA	-3.4	0.4	-0.8	-0.5	-5.8	-4.3	-1.6	-2.8	-10.5	-6.1	-4.6	-3.0
AT	3.1	7.7*	7.0*	3.3*	0.0	2.3	-0.8	-7.9	0.6	11.0	-0.9	-14.7
SW	9.6	-0.9	1.8*	4.5*	10.4	1.5	3.6	5.9	8.4	-1.3	2.5	3.3
SA	10.0	0.5	4.4*	-1.8*	8.8	7.6	1.7	-2.6	23.8	17.5	9.4	1.1
AT	2.3	7.9	6.6	2.2	-1.0	2.6	-1.3	-9.6	-0.4	11.8	-1.5	-15.8
DA	6.0	-0.1	0.7	3.4	5.4	-2.1	1.7	3.6	1.7	-6.5	-0.6	1.4
SA	11.2	0.3	4.6	-2.0	10.9	7.9	2.5	-2.9	26.5	17.1	10.4	0.8
SW	10.1	-1.4	0.6	4.4	15.2	3.6	5.1	9.4	16.7	3.7	6.1	7.6
DA	-3.1	0.4	-0.4	-0.6	-5.7	-3.9	-1.7	-3.4	-10.2	-7.4	-4.6	-4.0
SA	8.7	0.2	2.9	-2.6	8.8	7.2	1.8	-1.2	23.6	16.1	9.5	4.0
AT	3.1	7.7	7.1	3.3	0.1	2.8*	-0.7*	-7.7*	0.8	11.5*	-0.6	-14.3*
SW	12.2	-1.1	2.4	5.2	15.2	3.7*	4.8*	8.3*	17.3	3.2*	6.0*	5.7*
DA	-3.1	0.4	-0.9	-0.8	-5.7	-4.0*	-1.6*	-3.0*	-10.3	-7.1*	-4.5*	-3.0*
SA	10.0	0.5	4.4	-1.9	8.8	7.4*	1.7*	-2.8*	23.9	16.2*	9.4*	1.1

Units are percentages, except in the case of accumulated time, with units of percentage per hour. Asterisks indicate coefficients of the best model that are significantly different from zero. For example, for the model including AT, SW, DA, and SA, there is a 3.3 % increase per hour during N3 sleep for physiological ripples, and a decrease of 7.7 % and 14.3 % per hour for pathological

ripples and fast ripples respectively. Legend. AT: accumulated time; SW: slow wave amplitude; DA: delta band activity; SA: sigma band activity; ND: non-discriminated sleep stages.

Supplementary Table S4. Akaike Information Criterion (AIC) value, relative to the model with constant spread during the whole investigated period

	Physiological ripples		Pathological ripples		Fast ripples	
	ND	D	ND	D	ND	D
Const.	0.0	-11.7*	0.0	-163.2	0.0	-79.3
AT	1.8	-6.2	-36.0	-162.3	-8.5	-75.3
SW	-13.3*	-7.1	-107.5	-167.9	-10.7	-74.5
DA	-14.2*#	-9.2*	-61.0	-163.2	-4.9	-76.4
SA	-7.6	-3.0	-140.4	-180.8*	-93.2*	-87.3
AT+SW	-12.9*	-1.6	-108.6	-164.6	-11.8	-70.0
AT+DA	-12.5*	-4.2	-71.2	-161.5	-8.9	-72.4
AT+SA	-5.9	1.3	-141.7	-178.3*	-90.4*	-84.1
SW+DA	-13.1*	-2.4	-108.3	-168.8	-9.2	-71.9
SW+SA	-12.7*	1.7	-179.1*	-180.8*	-95.7*#	-80.5
DA+SA	-13.7*	-0.6	-156.8	-181.1*	-90.9*	-83.3
AT+SW+DA	-12.4*	4.6	-109.1	-166.3	-9.9	-67.4
AT+SW+SA	-12.1*	6.2	-177.1*	-177.0*	-93.7*	-82.5
AT+DA+SA	-11.6*	3.0	-155.2	-178.1*	-88.1	-81.1
SW+DA+SA	-12.2*	2.9	-179.7*	-181.3*#	-91.3*	-79.5
AT+SW+DA+SA	-9.9*	8.7	-177.7*	-177.4	-89.3	-71.4

Lower AIC values indicate preferred models. A difference greater than 6 between two models is necessary for statistical significance. Asterisks indicate models that are not significantly different from the best model, indicated by a number sign.

Legend. D, discriminated sleep stages; DA: delta band activity; SA: sigma band activity; ND, non-discriminated sleep stages; AT: accumulated time; SW: slow wave amplitude.

Supplementary Table S5. Coefficients of the relative spread variation for the tested models

	Physiological ripples				Pathological ripples				Fast ripples			
Stage	ND	REM	N2	N3	ND	REM	N2	N3	ND	REM	N2	N3
Variable		-4.2	0.4	3.0		-13.9*	1.4*	10.5*		-6.5	1.3	2.7
AT	-0.3	-0.4	0.7	0.5	-2.4	1.2	-1.7	-4.5	-0.8	0.8	-0.4	-2.6
SW	1.9	-0.1	0.2	1.4	7.2	1.1	2.7	2.8	2.1	-0.4	0.6	0.9
DA	1.8*	0.7	0.6	1.8	5.4	-1.5	1.7	1.9	1.4	-0.7	0.0	0.4
SA	2.0	0.5	0.6	0.0	7.8	4.2	3.5	1.4	3.7	1.9	1.8	1.7
AT	0.2	-0.4	0.9	0.9	-0.7	1.4	-0.9	-3.7	-0.4	0.8	-0.3	-2.4
SW	2.1	-0.1	0.5	1.5	6.6	1.2	2.5	2.5	1.8	-0.4	0.5	0.7
AT	0.0	-0.4	0.9	0.7	-1.5	1.2	-1.4	-4.3	-0.6	0.9	-0.4	-2.6
DA	1.9	0.7	0.8	1.8	4.5	-1.5	1.5	1.8	0.9	-0.7	-0.1	0.3
AT	0.1	-0.2	1.0	0.5	-0.9	2.4	-0.8	-4.1	-0.1	1.4	0.1	-1.6
SA	2.1	0.5	0.8	0.1	7.2	4.4	3.3	0.5	3.6	2.0	1.8	1.3
SW	1.2	-0.7	-0.4	-0.1	8.9	2.8	3.1	3.6	3.2	0.0	1.2	1.7
DA	0.8	1.1	0.9	1.8	-2.0	-3.1	-0.6	-1.0	-1.4	-0.7	-0.9	-1.0
SW	1.3	-0.2	0.0	1.4	4.8	0.4	1.7	2.9	0.7*	-0.8	-0.1	1.0
SA	1.5	0.5	0.6	0.0	5.8	4.1	2.8	1.5	3.4*	2.0	1.8	1.7
DA	1.3	0.7	0.5	1.8	3.2	-1.7	0.9	2.1	0.1	-0.8	-0.5	0.5
SA	1.6	0.4	0.5	0.2	6.8	4.3	3.2	1.6	3.6	1.9	1.9	1.7
AT	0.2	-0.5	0.9	0.7	-0.7	1.8	-0.9	-3.6	-0.4	0.9	-0.2	-2.3
SW	1.4	-0.7	-0.1	0.0	8.3	2.9	2.8	3.0	2.9	0.0	1.2	1.3
DA	0.8	1.1	0.8	1.8	-1.9	-3.1	-0.5	-0.7	-1.3	-0.7	-0.9	-0.8
AT	0.4	-0.3	1.0	1.2	0.0	2.5	-0.4	-2.9	0.0	1.4	0.1	-1.2
SW	1.5	-0.2	0.3	1.5	4.7	0.5	1.6	2.6	0.7	-0.7	-0.1	0.9
SA	1.6	0.5	0.8	0.3	5.8	4.3	2.8	0.9	3.4	2.1	1.9	1.4
AT	0.3	-0.2	1.1	1.0	-0.5	2.5	-0.7	-3.6	-0.1	1.5	0.1	-1.5
DA	1.5	0.7	0.6	1.8	2.9	-1.7	0.8	1.9	0.1	-0.9	-0.5	0.4
SA	1.7	0.4	0.7	0.4	6.5	4.4	3.1	0.8	3.6	2.0	2.0	1.4
SW	0.5	-0.8	-0.6	-0.1	6.2	2.0*	2.0*	3.5*	1.8	-0.5	0.5	1.6
DA	0.9	1.1	0.9	1.9	-1.7	-2.8*	-0.5	-0.8	-1.3	-0.6	-0.9	-0.7
SA	1.5	0.6	0.6	0.2	5.8	4.0*	2.8*	1.5	3.4	2.0	1.8	1.7
AT	0.4	-0.4	1.0	1.0	0.0	2.8	-0.4	-2.9	0.0	1.5	0.2	-1.1
SW	0.8	-0.8	-0.4	0.0	6.2	2.1	1.9	3.1	1.8	-0.4	0.6	1.4
DA	0.8	1.1	0.8	1.8	-1.7	-2.9	-0.5	-0.6	-1.3	-0.7	-0.9	-0.7
SA	1.6	0.5	0.8	0.4	5.8	4.2	2.8	0.9	3.4	2.1	1.9	1.4

Units are percentages, except in the case of accumulated time, with units of percentage per hour. Asterisks indicate the coefficients of the best model that are significantly different from zero. For example, in the model including only the sleep stage, there is a 13.9 % decrease in spatial spread of pathological ripples during REM sleep, as opposed to a 10.5 % increase during N3 sleep.

Legend. AT: accumulated time; SW: slow wave amplitude; DA: delta band activity; SA: sigma band activity; ND: non-discriminated sleep stages.

SUPPLEMENTARY FIGURES

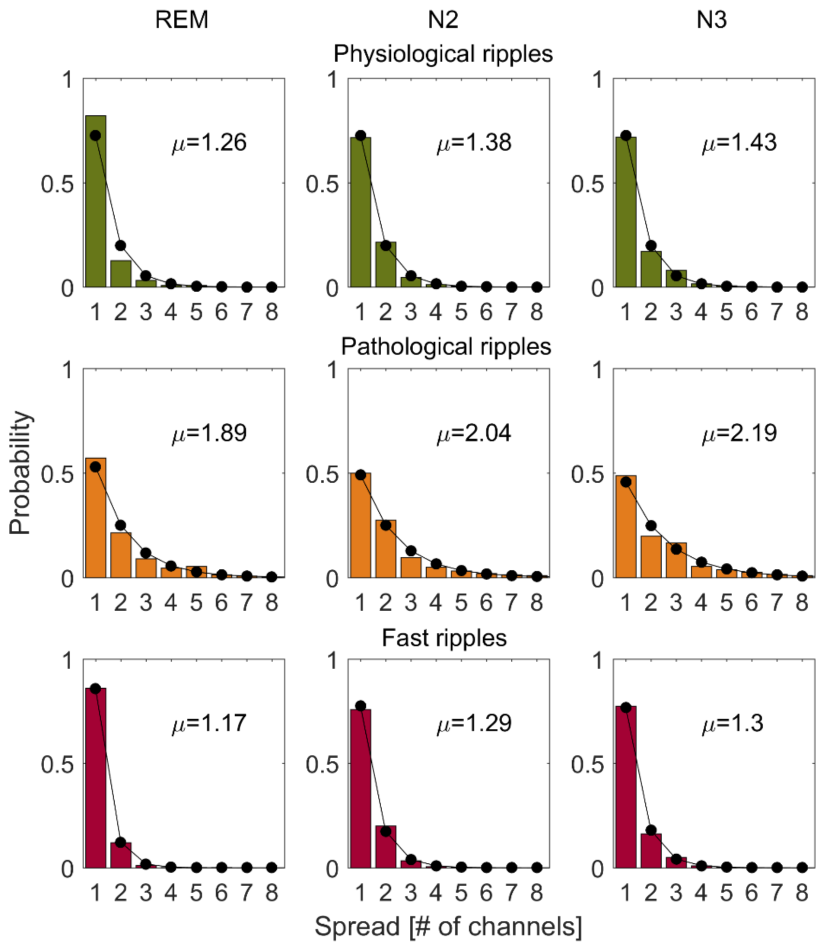

Supplementary Figure S1. Average spread of HFOs for all patients across different sleep stages. The color bars represent the measured spread and the black dots, the values fitted by the Geometric model with the mean spread μ indicated in the panels. It can be seen, that the model adjusts very well to the measurements in all cases. For all types of HFOs (physiological ripples, pathological ripples, fast ripples), the spread is largest in N3 sleep and lowest in REM sleep. The pathological ripples have a larger spread than the physiological ripples, and the fast ripples have the lowest spread overall.
