# Supplementary material for: Physiological and pathological high-frequency oscillations have distinct sleep-homeostatic properties
Source: Neuroimage Clin. 2017 Feb 24;14:566–73. doi: 10.1016/j.nicl.2017.02.018 (PMC5349616; doi:10.1016/j.nicl.2017.02.018)
Supplement: Supplementary file 2 — Mathematical and statistical models. [file mmc2.pdf]

Supplementary file: Mathematical and statistical models

N. von Ellenrieder, F. Dubeau, J. Gotman, and B. Frauscher

Manuscript: Physiological and Pathological High-Frequency Oscillations have distinct Sleep-Homeostatic Properties

In this supplementary document we give a detailed description of the models adopted for the High Frequency Oscillation (HFO) rate and spatial spread, and the model comparison approach used for statistical testing.

1 Rate:

1.1 Model

A Poisson process was adopted for modeling the occurrence of HFOs. This process models events that cannot overlap in time, and in which the probability of occurrence is independent from the time elapsed since the last occurrence. Since we are modeling the *subject-level* HFOs, i.e. we count any simultaneous events in any set of channels as a single event, the data does not have overlapping HFOs, and the Poisson process is an appropriate model.

The probability function of the number of events of a Poisson process occurring in a given time interval is given by

$$P\{x = k\} = \frac{\lambda^k}{k!} \mathrm{e}^{-\lambda}, \tag{1}$$

where x is the observed number of events, and λ is the parameter of this Poisson distribution, i.e. the rate of events per unit time.

To model our problem, we divide the EEG recordings in intervals of 30 s duration, and count the number of HFOs in each of these intervals. The 30 s segments are selected to coincide with the epochs of the sleep scoring. The HFO rate in each of the 30 s segments is modeled as a fixed value associated to the patient, with relative variations given by a linear combination of predictor variables

$$\lambda_{n,m} = \lambda_m \left(1 + \sum_{i=1}^{3} I_{i,n,m} (\alpha_{stage,i} + \alpha_{AT,i} t_{n,m,i} + \alpha_{DA,i} \delta_{n,m,i} + \alpha_{SW,i} s_{n,m,i}) \right) \tag{2}$$

where $\lambda_{m,n}$ is the HFO rate in segment n of patient m, λ_m is the average rate in all investigated segments of patient m, $I_{i,n,m}$ is an indicative variable that takes value 1 if the segment n of patient m is in the i-th sleep stage, with $i = 1$ for REM sleep, $i = 2$ for N2 sleep, and $i = 3$ for N3 sleep. The variable $t_{n,m,i}$ is the accumulated time spend in sleep stage i at segment n for patient m. The variable $\delta_{n,m,i}$ is the delta band RMS amplitude at segment n for patient m, when the sleep stage is i, and zero otherwise. Similarly, $s_{n,m,i}$ is the average amplitude of sleep slow waves in segment n for patient m, when the sleep stage is i, and zero otherwise. The coefficients $\alpha_{stage,i}, \alpha_{AT,i}, \alpha_{DA,i}$, and $\alpha_{SW,i}$ indicate the degree to which the sleep stage, accumulated sleep time, delta band activity, and slow wave amplitude affect the average HFO rate in the patient cohort. Note that there is a maximum of twelve α coefficients if all the variables are included in the model, plus one λ_m coefficient for each patient. In cases in which the sleep stages are not included in the model, expression (2) simplifies to

$$\lambda_{n,m} = \lambda_m (1 + \alpha_{AT} t_{n,m} + \alpha_{DA} \delta_{n,m} + \alpha_{SW} s_{n,m}), \tag{3}$$

with a maximum of three α coefficients.

An important point is that the predictor variables accumulated sleep time, delta band activity, and slow wave amplitude are transformed so that in each sleep stage they have zero mean. The delta band activity and slow wave amplitude variables are also normalized to have unit variance in each sleep stage, in order to make them independent of the measuring units and of equal importance. The accumulated time is expressed in hours, which also leads to a variance close to one.

Note that the coefficients of the predictor variables are constant for all the patients, i.e. while the average HFO rate is assumed to be patient dependent, the relative variation due to the predictive variables are supposed to explain common phenomena of the whole cohort.

Writing (3) in a compact matrix notation

$$\bar{\lambda} = \bar{\lambda_m} \circ (1 + X\bar{\alpha}), \tag{4}$$

where $\bar{\lambda}$ is the vector formed by the concatenation of the HFO rate in every segment of every subject, $\bar{\lambda_m}$ is a vector of the same size as $\bar{\lambda}$ formed by the average HFO rate in each subject (λ_m) repeated for each segment of the corresponding subject, $\circ$ is the Hadamard product (i.e. one to one element-wise product), X is a matrix where each column corresponds to a different predictor variable and each row is formed by the value of the predictor variables in segment n of patient m. Finally, $\bar{\alpha}$ is the concatenation of the coefficients associated to the different predictor variables.

Then, the joint probability of all the observations can be written as

$$P\{x_{n,m} = k_{n,m}\} = \prod_{n=1}^{N} \prod_{m=1}^{M} \frac{\lambda_m^{k_{n,m}} (1 + \bar{\alpha}^T \bar{x}_{n,m})^{k_{n,m}}}{k_{n,m}!} \mathrm{e}^{-\lambda_m (1+\bar{\alpha}^T \bar{x}_{n,m})}, \tag{5}$$

where $k_{n,m}$ is the number of HFOs observed in time segment n of patient m, and $\bar{x}_{n,m}$ is the row of matrix X corresponding to the value of the predictor variables in segment n for patient m.

1.2 Parameter estimation:

In this section we find the maximum likelihood (ML) estimators of the parameters of the distribution (i.e. λ_m and $\bar{\alpha}$). The log-likelihood function is given by

$$L(\lambda, \alpha) = \sum_{m=1}^{M} \sum_{n=1}^{N_m} \left[-\ln(k_{n,m}!) + k_{n,m} \ln\lambda_m + k_{n,m} \ln\left(1 + \bar{\alpha}^T \bar{x}_{n,m}\right) - \lambda_m \left(1 + \bar{\alpha}^T \bar{x}_{n,m}\right)\right]. \tag{6}$$

By taken the derivative with respect to λ_m and equating to zero, we get

$$\lambda_m + \lambda_m \bar{\alpha}^T \frac{1}{N_m} \sum_{n=1}^{N_m} \bar{x}_{n,m} = \frac{1}{N_m} \sum_{n=1}^{N_m} k_{n,m}. \tag{7}$$

By construction all the predicting variables have zero mean, then $\sum_{n=1}^{N_m} \bar{x}_{n,m} = \bar{0}$, and (7) reduces to

$$\lambda_m = \frac{1}{N_m} \sum_{n=1}^{N_m} k_{n,m}, \tag{8}$$

showing that the estimates of the λ_m parameters are decoupled from $\bar{\alpha}$.

Once the estimates of λ_m are known, the ML estimate of $\bar{\alpha}$ can be obtained by maximizing (6), with a numerical optimization method. We performed an unconstrained minimization with a quasi-Newton method.

2 Spread:

2.1 Model

For the spread we adopted a model based on a Geometric Distribution. In this model, the difference in the probability of an HFO occurring simultaneously in $j+1$ channels compared to j channels is independent of j. This model is just an approximation to the real distribution, since in theory the model would allow simultaneous HFOs in an arbitrarily large number of channels, albeit with almost null probability. However, the approximation provided by this model is very good, as can be seen in Supplementary Figure S3.

The probability of an HFO being simultaneously detected in j channels is then given by

$$P\{y=j\} = \mu^{-1}(1-\mu^{-1})^{j-1}, \tag{9}$$

where y is the observed number of involved channels and μ is the mean number of channels in which an HFO is simultaneously detected, i.e. the mean spread.

Again, we base our model on a fixed mean spread, with a patient dependent average value and relative variations common to the whole cohort

$$\mu_{n,m} = \mu_m \left(1 + \sum_{i=1}^{3} I_{i,n}(\beta_{stage,i} + \beta_{AT,i} t_{n,m,i} + \beta_{DA,i}\delta_{n,m,i} + \beta_{SW,i} s_{n,m,i})\right), \tag{10}$$

where $\mu_{m,n}$ is the mean HFO spread in segment n of patient m, μ_m is the average spread in all investigated segments of patient m, the coefficients $\beta_{stage,i}, \beta_{AT,i}, \beta_{DA,i}$, and $\beta_{SW,i}$ indicate the degree to which the sleep stage, accumulated sleep time, delta band activity, and slow wave amplitude affect the average HFO spread in the patient cohort. Or, in the case of models not taking into account the sleep stage

$$\mu_{n,m} = \mu_m(1 + \beta_{AT} t_{n,m} + \beta_{DA}\delta_{n,m} + \beta_{SW} s_{n,m}). \tag{11}$$

This can be written in a compact matrix notation as

$$\bar{\mu} = \bar{\mu_m} \circ (1 + X\bar{\beta}), \tag{12}$$

where $\bar{\mu}$ is the vector form by the concatenation of the mean HFO spread in every segment of every subject, $\bar{\mu_m}$ is a vector of the same size as $\bar{\mu}$ formed by the average HFO spread in each subject (μ_m) repeated for each segment of the corresponding subject, and $\bar{\beta}$ is the concatenation of the coefficients associated to the different predictor variables.

The joint probability function for all the observations can be written then as

$$P\{y_{n,m} = j_{n,m}\} = \prod_{n=1}^{N}\prod_{m=1}^{M} \mu_m^{-1}(1+\bar{\beta}^T \bar{x}_{n,m})^{-1}\left[1 - \mu_m^{-1}(1+\bar{\beta}^T\bar{x}_{n,m})^{-1}\right]^{j_{n,m}-1}, \tag{13}$$

where $j_{n,m}$ is the spread observed for segment n of patient m.

2.2 Parameter estimation

To obtain the ML estimator of the distribution parameters μ_m and $\bar{\beta}$ we start with the log-likelihood function

$$L(\mu,\beta) = \sum_{m=1}^{M}\sum_{n=1}^{N_m}\left\{\ln\left[\mu_m\left(1+\bar{\beta}^T\bar{x}_{n,m}\right)\right] + c_{n,m}\ln\left[1-\mu_m^{-1}\left(1+\bar{\beta}^T\bar{x}_{n,m}\right)\right]\right\}. \tag{14}$$

where $c_{m,n}$ is the observed average spread in each 30 s segment. By maximizing with respect to μ_m, we get

$$(\mu_m - 1) + \mu_m\bar{\beta}^T\frac{1}{N_m}\sum_{n=1}^{N_m}\bar{x}_{n,m} = \frac{1}{N_m}\sum_{n=1}^{N_m}(c_{n,m}-1). \tag{15}$$

Again, the fact that the mean value of the predicting variables is null leads to a decoupling of the $\bar{\beta}$ parameters, yielding

$$(\mu_m - 1) = \frac{1}{N_m} \sum_{n=1}^{N_m} (c_{n,m} - 1). \tag{16}$$

Once the estimates of μ_m are known, the ML estimate of $\bar{\beta}$ can be obtained by maximizing (6), with a numerical optimization method. We performed an unconstrained minimization with a quasi-Newton method.

3 Model Selection: Akaike Information Criterion

We compare models that include different subsets of the predictor variables, and use the Akaike Information Criterion (AIC) to select the best model. A classical approach for selecting the best model would be to test for coefficients significantly different from zero, e.g. for nested Gaussian models a classical model selection would imply F-tests on the coefficients. The AIC is completely equivalent to F-tests in the case of nested Gaussian models, but it can handle non-nested, non-Gaussian models as well. Just as in the case of classical tests, the AIC looks for a balance between goodness-of-fit and simplicity, the latter measured by the number of parameters in the model in the case of AIC, and related to the number of degrees-of-freedom in classical tests.

The AIC value of any model is given by

$$\mathrm{AIC} = 2k - 2L(\bar{\theta}), \tag{17}$$

where k is the number of parameters in the model, $L(\cdot)$ is the log-likelihood function of the model, and $\bar{\theta}$ is a vector with the k ML estimates of the parameters. When comparing models, the best model is the one with the lowest AIC value.

When comparing different models, there will always be a 'best model', i.e. one of the competing models will have the lowest AIC value. However, there is randomness in the measurements, that affects the value of the likelihood function, and leads to randomness in the AIC value even if computed for data generated by the same model. Hence, it is convenient to determine whether a model is significantly better than another from a statistical point of view. This can be done by comparing the AIC values by computing the relative likelihood of the models

$$\mathrm{RL}_{i,j} = e^{\left(\mathrm{AIC}_i - \mathrm{AIC}_j\right)/2}. \tag{18}$$

If the relative likelihood between two models is larger than 20 times, there will a probability lower than 0.05 of selecting the wrong 'best' model through the use of AIC. This relative likelihood of 20 corresponds to a difference of 6 in AIC values.

To perform a selection between two models, first the AIC value of each model must be computed, and the model with lowest AIC value is considered significantly better if the difference in AIC values is larger than six. The expression of the log-likelihood function to be used can be found in the previous sections, and should be evaluated at the parameter values obtained as ML estimates.
